# Association of depression with treatment outcomes in Type 2 Diabetes Mellitus: A cross-sectional study from Karachi, Pakistan

**DOI:** 10.1186/1471-244X-11-27

**Published:** 2011-02-15

**Authors:** Saman I Zuberi, Ehsan U Syed, Junaid A Bhatti

**Affiliations:** 1 Aga Khan University Hospital, Karachi, Pakistan; 2 Douglas Mental Health University Institute, Verdun, Canada

## Abstract

**Background:**

To assess the associations of depression with glycemic control and compliance to self-care activities in adult patients with Type 2 Diabetes Mellitus

**Methods:**

This cross-sectional study was conducted at a tertiary-care hospital in Karachi (Aga Khan University Hospital). Equal numbers of depressed and non-depressed patients were consecutively recruited from the diabetic clinic. Information on demographic and clinical characteristics was collected in face-to-face interviews and from medical records. Hospital Anxiety Depression Scale (HADS) was used to measure depression. Associations of depressed status (HADS ≥ 8) with poor glycemic control (Hemoglobin A1c level ≥ 7%) and compliance to self-care activities were assessed by logistic regression analyses.

**Results:**

A total of 286 patients were included in this study with a male-female ratio of 1.2:1. Mean age was 52 years and in 64.7% of them, the duration of diabetes was more than 3 years. Depressed patients were more likely to be female (adjusted odds ratio [OR] = 1.88; 95% confidence interval [95%CI] = 1.07-3.31), had a family history of diabetes (OR = 2.64; 95%CI = 1.26-5.55), and poor glycemic control (OR = 5.57; 95%CI = 2.88-10.76) compared with non-depressed patients. Depression was also associated with low compliance to self-care activities such as taking dose as advised (OR = 0.32; 95%CI = 0.14-0.73), dietary restrictions (OR = 0.45; 95%CI = 0.26-0.79) and foot care (OR = 0.38; 95%CI = 0.18-0.83).

**Conclusions:**

Adult patients with Type 2 Diabetes who have depression were more likely to have poor glycemic control and lower compliance to self-care activities, and they might need particular attention during follow-up visits.

## Background

Diabetes and obesity are now seen as a global epidemic; with high figures from South Asia [1]. The World Health Organization has estimated that in 2030, half of the 333 million people living with diabetes will be from Asia alone [2]. Pakistan is an Asian country bordering the Arabian Sea with a population of over 176 million as estimated in July 2009 [3]. The epidemic of diabetes is particularly relevant to Pakistan. Surveys from different parts of the country have estimated a prevalence of 6% in men and 3.5% in women living in urban areas [4].

Self-care is critical to outcome of diabetes and includes compliance to medication, diet modifications, and regular exercise [5]. If any of these are compromised, the target glycemic control may not be achieved [6,7]. Studies of the diabetic population in Pakistan showed that more than two-thirds of investigated samples had poor glycemic control [8-10]. Reasons for these treatment outcomes have not been investigated before.

The international research showed that depression was associated with poor compliance to treatment [11-13]. In particular, depressed mood in diabetic patients might lead to pessimism regarding perceived benefits and lowered self-efficacy, and could result in poor self-care and compliance [14]. Furthermore, it was clearly indicated that depression in Type 2 Diabetic patients could severely impact management of diabetes through a higher symptom burden [15], increased functional impairment [15], poorer glycemic control [16], and more diabetic complications [17,18]. Finally, the course of depression is also more chronic and severe in people with diabetes [19].

A systematic review of mental health studies from Pakistan showed that mean overall prevalence of anxiety and depressive disorders in the community population was 34% [20]. Considering this high prevalence of depression in Pakistani population, its influence on treatment outcomes of chronic medical conditions has rarely been investigated [10,20]. In this study we assessed if any associations existed between depression, glycemic control, and compliance with self-care activities in the patients attending diabetic clinics in Pakistan.

## Methods

### Setting

The Aga Khan University Hospital is a 500-bedded tertiary care hospital that caters mostly to middle and upper socio-economic strata. This study was conducted in the specialist endocrine clinics. The American Diabetic Association (ADA) recommendations of diet, exercise, glycemic monitoring, and investigations are followed on all patients [5]. A nurse specially trained in teaching methods of self-care (monitoring blood glucose, administration of insulin, timings of medicines, diet, and exercise suggestions) is employed at the clinic and sees all patients.

### Study design

The study was conducted from August 2008 to June 2009. All consecutive patients having type 2 diabetes and its related complications between 18 and 60 years of age who had at least 2 clinic visits for management of diabetes mellitus were selected. Exclusion Criteria were those having history of psychiatric illnesses other than depression, and who were currently on anti-depressant treatment and/or those who had history of use of psychotropic drugs. Equal numbers of depressed and non-depressed patients were consecutively included in this comparative cross-sectional study. The sample size was calculated for each group to be 143; keeping beta error at 0.2, significance level 0.05 and with the percentage difference in poor glycemic control between the groups assumed to be 17% as determined in a systematic review by Lustman and colleagues [21].

### Measures

Nursing assessment was performed for each participant that included recording of blood pressure, height and weight. After an informed consent, a semi-structured clinical interview was performed by the main investigator before each participant's consultation with the endocrinologist. Privacy was maintained throughout the interview. Details of each participant's demographics, history of psychiatric illness/exposure to psychotropic medications, family history of diabetes/depression, and compliance to self-care activities were recorded.

Medical records were examined for presence of diabetes-related complications; renal ( "proteinuria", "microalbuminuria" and "renal failure"), ophthalmic ("retinopathic changes" and "cataract"), neurological ("stroke" or "transient ischemic attack"), dyslipidemia ("Deranged lipids"), cardiac ("Angina" or "myocardial infarction"), hypertension, arthropathies ("Charcot joints" or "diabetes-related joint problem"), and neuropathies.

Depression was assessed using the 7-item depression subscale of Hospital Anxiety Depression Scale (HADS) [22]. HADS is a widely used scale validated in the local language (Urdu) with good sensitivity and specificity [23]. Somatic symptoms of depression (e.g. insomnia and weight loss) are not part of this scale and therefore findings were not confounded by symptoms of uncontrolled diabetes. A summative score is calculated (range 0-21) with cut-off point for depression at 8 [24].

Glycosylated Hemoglobin (HbA1c) was used as a measure of glycemic control (<7% good compliance; ≥7% poor compliance). The sample HbA1c level was measured within next 6 months by using few drops of blood on High-Performance Liquid Chromatography (HPLC). It is accepted as the best measure and is used to guide clinical management [5].

Compliance to self-care activities was measured using a self-report technique inquiring on treatment (timing/dosing), diet, exercise, and care of feet. Each participant was asked about each self-care activity with the following question in Urdu: "What percentage of the time do you think you have followed the doctor's recommendations?" Compliance was recorded as "poor" if their reply was less than 50% of the time and "good" if it was 50% or more. Study methods were approved from the Aga Khan University Ethics Research Committee.

### Statistical analysis

Frequencies of all demographic and clinical variables were computed to identify sample characteristics. Body Mass Index (BMI) was computed using standard formula. Quantitative variables such as age, duration of illness, and BMI were regrouped in three equally distributed categories. Associations of above mentioned variables with depressed status (HADS ≥ 8) and glycemic control were assessed using chi-square or t-test. Further, a multivariate logistic regression model was constructed including all significantly associated variables (P < 0.05) with backward selection strategy. Statistical Package for Social Sciences Version 15.0 was used for these analyses.

## Results

Information on all participants (N = 286) was available. The proportion of women (N = 158, 55.2%) in study sample was slightly more than men (N = 128, 44.8%), and depression was significantly higher in women than men (60.8% vs. 39.2%; P = 0.03). All participants were aged between 31 to 60 years. Majority (81.1%) of them had a family history of diabetes. Family history of depression was reported by 26.2% of them with no difference between genders. One in five patients (N = 60, 21.0%) was currently smoking cigarettes; most were men (N = 47, 78.3%). Over two-third of patients (N = 205, 71.6%) had poor glycemic control.

Mean BMI value was 29.6 kg/m^2^, (Standard deviation [SD] = 4.7). Women had significantly higher BMI values than men (30.4 kg/m^2^, SD = 5.2 vs. 28.6 kg/m^2^, SD = 3.8; *P *= <0.01). BMI was significantly higher in those with poor glycemic control (29.2 kg/m^2 ^vs. 27.2 kg/m^2^; *P *< 0.01).

Majority (93.7%) of the patients were on oral hypoglycemic agents; 63.3% on monotherapy and a further 30.4% on combination with insulin. Poor glycemic control was significantly higher in patients on insulin and combination therapy than those on oral hypoglycemics only (86.6% vs. 63.0%; *P *< 0.01). Most patients (64.7%) had duration of illness of more than three years. Those with poor glycemic control had significantly longer duration of diabetes (8.2 vs. 6.1 years; *P <*0.01). Almost four out of five patients (N = 234, 81.8%) had a diabetes-related complication. Over half of the patients practiced self-care activities except exercise, followed by only 28.0% of them, and women significantly less than men (22.2% vs. 35.2%; *P *= 0.02). Those with poor glycemic control were following exercise recommendations significantly less than those with good glycemic control (24.4% vs. 37.0%; *P *= 0.03).

Depressed patients reported more family history of depression (32.2% vs. 20.3%) and diabetes (88.8% vs. 73.4%) than non-depressed patients. Similarly, more depressed patients reported a diabetes-related complication than non-depressed patients (87.0% vs. 77.0%; *P *= 0.03). Monotherapy was significantly higher in the depressed group (62.9% vs. 37.1%; *P *= 0.02) compared with non-depressed patients.

Being a female and a family history of diabetes was significantly associated with depression (Table 1). Similarly, depressed patients were more likely to have poor glycemic control than non-depressed patients. The mean HbA1c level was significantly higher in depressed than non-depressed patients (8.5% vs. 7.7%; p < 0.001). Moreover, depressed patients were more likely to not to take dose as advised, follow dietary restrictions, or perform foot care as compared with non-depressed patients.

**Table 1:** Characteristics of patients presenting to endocrine clinic at the Aga Khan Hospital of Karachi (N = 286)

	Depressed		Not depressed		*P*	Adjusted Odds ratio	95% CI
					
	N	%	N	%			
Gender					0.03		
- Men	56	39.2	72	50.3			
- Women	87	60.8	71	49.7		1.88	1.07-3.31
Age (y)					0.92		
- 31-47	41	28.7	44	30.8			
- 48-56	54	37.8	53	37.1			
- 57-60	48	33.6	46	32.2			
Family history: depression	46	32.2	29	20.3	0.02		
Family history: diabetes	127	88.8	105	73.4	0.01	2.64	1.26-5.55
Smoker	35	24.5	25	17.5	0.15		
Body Mass Index (kg/m²)					0.02		
- 18-<25	11	7.7	26	18.2			
- 25 - <30	70	49.0	68	47.6			
- >30	62	43.4	49	34.3			
Duration of illness (y)					0.04		
- 0-3	42	29.4	59	41.3			
- 4-10	62	43.4	43	30.1			
- >10	39	27.3	41	28.7			
Complications							
- Renal	37	25.9	20	14.0	0.01		
- Ophthalmic	37	25.9	23	16.1	0.04		
- Cerebrovascular	22	15.4	6	4.2	0.001		
- Hypertension	9	6.3	10	7.0	0.81		
- Dyslipidemia	89	62.2	84	58.7	0.55		
- Cardiac	35	24.5	19	13.3	0.01		
- Arthropathies	17	11.9	12	8.4	0.33		
- Neuropathies	43	30.1	30	21.0	0.08		
Treatment					0.04		
- Oral hypoglycemic	81	56.6	100	69.9			
- Insulin	9	6.3	9	6.3			
- Insulin & oral hypoglycemic	53	37.1	34	23.8			
Compliance of self care activities*							
- Taking dose on time	115	80.4	125	87.4	0.11		
- Taking dose as advised	105	73.4	132	92.3	0.01	0.32	0.14-0.73
- Dietary restrictions	74	51.7	101	70.6	0.01	0.45	0.26-0.79
- Exercise	28	19.6	52	36.4	0.002		
- Foot care	105	73.4	129	90.2	<0.001	0.38	0.18-0.83
Poor glycemic control (Hb_1Ac _≥ 7)	124	86.7	81	56.6	<0.001	5.57	2.88-10.76

* Following recommendations 50% or more of the time

## Discussion

Results of this study clearly showed that depression was associated with poor treatment outcomes in included patients. For instance, depressed patients had significantly poorer glycemic control than non-depressed patients. Results further indicated that this poor outcome could be explained by lower compliance to the recommended self-care activities such as taking dose on time, dietary restrictions, and foot care.

Before data collection of this study, we found only two studies from Pakistan that assessed mental health of diabetic patients. Both studies compared diabetic patients and with non-diabetic controls, and included 50 or fewer patients in each group [25,26]. Depression was found in greater proportion in the diabetic groups than non diabetics in both studies. A recent study by Khuwaja et al (2010) showed that 43.5% of patients attending diabetic clinics in Karachi had depression [27]. The study further indicated that blood pressure, fasting blood sugar, triglycerides, and BMI were associated with depression. However, measures of glycemic control and compliance to self-care activities were not included in that study. Our results clearly pointed out that information of treatment outcomes in depressed diabetic patients might help clinicians to better monitor such patients.

Our sample contained mostly patients with long duration of illness, diabetes-related complications, and less than half following self-care activities recommended by their endocrinologist. Results showed that majority of them had poor glycemic control which was consistent with previous findings in Pakistan showing that 78% of the diabetic patients had poor glycemic control [28]. The higher likelihood of poor glycemic control in the depressed patients than non-depressed patients as indicated in this study was consistent with findings from other international studies [21,29]. Moreover, our study indicated that depression was higher in patients with diabetes-related complications such as renal, cerebrovascular, cardiac and neuropathic and ophthalmic [30]. The possible explanation for above findings might be that the depression and psychological stress increase sympathetic nervous system activity, inflammatory, and platelet aggregation, and decrease insulin sensitivity, thereby contributing to poor glycemic control and increasing the risk of complications [31,32]. This suggested that regular evaluations of depression and glycemic control might be needed in diabetic clinics.

In our study, female gender was significantly associated with depression. It was previously shown that female gender was associated with depression in Type 2 Diabetes [33,34]. In a meta-analysis by De Groot et al, the combined prevalence of depression was significantly higher in Type 2 diabetic women than Type 2 diabetic men [17]. Our results showed that females were also less likely to follow recommendations of exercise and more likely to have greater BMI values. This suggested that women patients with Type 2 Diabetes Mellitus deserve special clinical attention in terms of ensuring that lifestyle changes were adopted by them.

Our study showed that depressed patients were less likely to follow necessary treatment recommendations such as taking proper doses, dietary restrictions, regular exercise, and foot care. Regression removed exercise from the final model however, other associations remained significant. This was consistent with previous findings of Lin et al showing that depression was associated with less physical activity, unhealthy diet, and low adherence to oral hypoglycemic, anti-hypertensive, and lipid-lowering medicines [12]. Another prospective cohort study showed that depression could predict lower medication adherence as well as adherence to dietary recommendations and exercise [35]. This suggested that more robust monitoring of depressed diabetic patients might be needed, perhaps by involving their family members in the process of care [36].

This study has several limitations. Firstly, the cross-sectional design is not without limitation since causality cannot be established. Although it may be argued that the possibility of selective loss of follow-up or non-random treatment changes is eliminated in such a design. Secondly, educational status and household income were not measured in this study although they have associations with both depression and diabetic patients with depression [35]. However participants can be considered to belong to middle or upper-middle socio-economic strata as they are coming to a specialty clinic and medical expenses are mostly borne out of pocket in Pakistan. Thirdly, self-report was used as a measure for self-care activities as no scale is validated in our population. Nevertheless, this method is highly sensitive to detection of non-adherence to medicines [38]. Lastly, generalization of study findings would require similar studies in other settings in Pakistan.

In conclusion, this study suggested that patients with type 2 diabetes should be screened for depressive symptoms, poor glycemic control, and non-compliance to self-care activities. Further, women and those who had a family history of diabetes should be closely watched for depressive symptoms. Finally, developing a valid instrument to measure compliance to self-care activities might be required to improve care in Pakistani diabetic clinics.

## Abbreviations

ADA: American Diabetic Association; BMI: body mass index; HADS, Hospital Anxiety Depression Scale; HbA1c: hemoglobin A1c; HPLC: high-performance liquid chromatography.

## Competing interests

The authors declare that they have no competing interests.

## Authors' contributions

SZ conceived the idea, made the research proposal, carried out the data collection, analysis and preparation of the first draft. ES supervised the formation of proposal, data collection and analysis. JAB was involved in the data analysis and revision of manuscript drafts. All authors read and approved the final manuscript.

## Author's information

SZ is a consultant psychiatrist who carried out this study under supervision during her residency training in psychiatry. ES is also a consultant psychiatrist who was her academic and clinical supervisor for this study. JAB is PhD in Public Health and was consulted for research statistics and analysis while at the university for his dissertation work.

## Pre-publication history

The pre-publication history for this paper can be accessed here:

http://www.biomedcentral.com/1471-244X/11/27/prepub
